# Monocyte Chemotactic Protein-1 (MCP1) Accumulation in Human Osteoclast Precursor Cultures

**DOI:** 10.3390/life12060789

**Published:** 2022-05-26

**Authors:** Nigel A. Morrison, Mark R. Forwood

**Affiliations:** School of Medical Science, Griffith University, Gold Coast Campus, Gold Coast, QLD 4215, Australia; m.forwood@griffith.edu.au

**Keywords:** monocyte chemotactic protein 1 (MCP-1), osteoclast, macrophage colony stimulating factor (M-CSF), granulocyte macrophage colony stimulating factor (GM-CSF), receptor activator of NF-κB ligand (RANKL), human CD14+ mononuclear cells

## Abstract

In vitro osteoclast methods require constant treatment with macrophage colony stimulating factor (M-CSF) to support precursor survival and addition of the differentiation agent receptor activator of NF-κB ligand (RANKL). Constant exposure to granulocyte macrophage colony stimulating factor (GM-CSF) suppresses human osteoclast formation in vitro. Addition of the chemokine monocyte chemotactic protein-1 (MCP1) to such cultures dramatically increases osteoclast formation and overcomes GM-CSF mediated suppression. We investigated the effect of M-CSF, GM-CSF and the combination of M-CSF and GM-CSF treatment on the expression of chemokines in human CD14+ cells in culture. Of assayed chemokines, MCP1 was the most abundant in terms of mRNA transcript and protein in M-CSF treated cultures and was suppressed by GM-CSF. MCP1 protein accumulated up to 50 ng/mL in culture medium, greatly exceeding other assayed chemokines. C-C chemokine receptor-2 (CCR2) is the receptor for MCP1: the formation of osteoclast-like cells was inhibited by constant exposure to the CCR2 antagonist RS102895, in part by decreasing expression of RANK, the receptor for RANKL.

## 1. Introduction

Chemokines are a diverse family of small secreted proteins, first identified via leukocyte chemotaxis, that mediate signalling between cells via G-coupled trans-membrane receptors [1,2,3]. Monocyte chemotactic protein-1 or MCP1 (also known as C-C motif chemokine ligand-2, CCL2) has effects that extend beyond chemotaxis and monocytes into many cell types and functions including bone remodelling via actions on osteoclasts [4]. Cellular and molecular actions of MCP1 were recently reviewed comprehensively [5]. Osteoclasts are responsible for bone resorption and form as giant multinucleated cells (MNC) by fusion of precursors derived from the monocyte-macrophage lineage, under the action of two factors: macrophage colony stimulating factor (M-CSF or CSF1) and receptor activator of NF-κB ligand (RANKL) [6,7]. The M-CSF is required for osteoclast precursor expansion and maintenance. RANKL is a member of the TNF-receptor super family and is expressed on osteoblasts and osteocytes in bone and is responsible for osteoclast differentiation. Although M-CSF is pro-osteoclast forming, granulocyte-macrophage colony stimulating factor (GM-CSF or CSF2) is anti-osteoclastic in that constant exposure to GM-CSF in the presence of M-CSF and RANKL strongly suppresses human osteoclast formation [8,9]. Suppression of osteoclast differentiation by GM-CSF treatment was reversed by adding high levels of exogenous MCP1, suggesting that MCP1 acts as a cell differentiation factor, determining a switch in cell fate in the presence of RANKL to either a multinucleated osteoclast or a mononuclear dendritic-like cell [9]. Exogenous MCP1 is a potent stimulator of multinucleation of osteoclasts and of increased osteoclast mediated bone resorption in the presence of RANKL [9]. Human osteoclast differentiation in vitro was blocked by both anti-MCP1 neutralising antibody [9] and a dominant negative truncated MCP1 (7ND) [10,11].

In mice, MCP1 knockout reduced osteoclast size [12], while knockout of MCP1 receptor CCR2 decreased osteoclast number and size in vivo [13]. Of clinical relevance, MCP1 is involved in parathyroid hormone (PTH) signalling; MCP1 is induced by PTH in rat bone [14] and MCP1 knockout animals have severely inhibited anabolic response to PTH due to reduced osteoclast numbers [15]. Monocyte chemotactic protein-1 is also an essential component of the catabolic effect of PTH through similar regulation of osteoclast numbers [16]. It is therefore involved in the normal formation of osteoclasts and their function in bone. In addition, MCP1 regulates the related but distinct multinucleated cell known as the foreign body giant cell (FBGC) [17,18]. During mouse and rat osteoclast formation macrophage inflammatory protein-gamma (also known as CCL9, or MIP-1γ) and its receptor CCR1 are highly induced and are functionally important for osteoclast differentiation [19,20,21]. No convincing CCL9 has been identified in the human and although CCL23 is considered a possible human ortholog it has only 38% identity with CCL9 [21] and was not considered in the present study. Human members of the MIP family, CCL3 (MIP1α) and CCL4 (MIP1β) are considered in this study.

The effect of M-CSF, GM-CSF and RANKL on the abundance of chemokines in human osteoclast precursor cultures is an important piece of information. In this paper we present data on CD14+ mononuclear cells purified from human blood, used as effective osteoclast precursors in order to further understanding of the biology of chemokines as they relate to human osteoclast differentiation.

## 2. Materials and Methods

### 2.1. Isolation and Culture of Human CD14+ Mononuclear Cells from Peripheral Blood

All cell culture reagents and vessels were from Life Technologies (Carlsbad, CA, USA). All peptide hormones were from Peprotech (Rehovot, Israel). Cells were routinely cultured in Dulbecco’s modification of Eagle’s medium (DMEM) augmented with 10% heat inactivated fetal bovine serum (FBS). Healthy volunteer donors gave blood samples with informed consent in protocols approved by the Griffith University Human Ethics Committee (approvals HREC50 and HREC3850). Blood was taken directly into BD Vacutainer CTP Cell preparation tubes (Becton Dickinson, Franklin Lakes, NJ, USA) that contain heparin to isolate peripheral blood mononuclear cells (PBMC) by centrifugation. Blood was centrifuged in a swing-out rotor for 15 min at 1500 relative centrifugal field (RCF) in a Beckman Allegra centrifuge (Beckman-Coulter, Brea, CA, USA). The mononuclear cell layer was removed and washed in 15 mL of phosphate buffered saline (PBS) and recovered by centrifugation at 300 RCF. Cells were counted using a hemocytometer slide and following the recommended amounts used for magnetic bead separation. The washed mononuclear fraction was incubated with magnetic-tagged anti-CD14 antibody and subsequently purified by magnetic selection to produce a CD14+ enriched fraction of PBMC according to the instructions of the manufacturer (MACS separation kit, Miltenyi Biotec, Bergisch Gladbach, North Rhine-Westphalia, Germany). Briefly, 20 μL of CD14 Microbeads were used per 10^7^ cells in a final volume of 100 μL. The suspension was incubated at 4C for 15 min and then cells recovered by centrifugation (300 RCF, 10 min) and washed. Cells were then resuspended and run through a column in a magnetic field. In this method, while the magnetic field is present, CD14+ cells are retained, and non-bound cells are washed through. After adequate depletion of the CD14- cells, the magnetic field was removed and CD14+ cells were collected. The yield of CD14+ cells was 18.9% (±4.5% standard deviation) of total PBMC measured from 13 different blood isolations.

CD14+ cells were cultured in plastic multi-well plates and in DMEM with 10% FBS augmented with various combinations of growth factors, essentially as described in Day et al. [8]. Cells were treated with either 40 ng/mL RANKL and 25 ng/mL M-CSF to produce osteoclast-like cells or 25 ng/mL M-CSF to produce macrophage-like cell cultures. GM-CSF was used at 25 ng/mL either alone or in combination with M-CSF. In dose response experiments, data are presented as fold change over untreated control of the same time of culture.

For experiments involving assays of cell culture medium, CD14+ mononuclear cells were treated with either M-CSF, GM-CSF and RANKL, as duplicate treatments, in various combinations in a pre-treatment or post-treatment condition in 6 well plates at 2 × 10^5^ cells/cm^2^ with 5 mL medium and cultured for several days. Medium (100 μL) was taken without replacement for assays at time points. Some experiments with pre-treatment and post-treatment were conducted in 12-well plates with 1 mL of medium and CD14+ cells plated at 2 × 10^5^ cells/well. Culture medium samples were assayed using BioRad Bioplex Human Cytokine 27-plex Immunoassay for chemokines and cytokines according to the manufacturer’s instructions (Bio-Rad, Hercules, CA, USA). Bioplex is a Luminex (Austin, TX, USA) immunobead based multiplex assay system that provides simultaneous assays of multiple target molecules with a high dynamic range and appropriate calibration tools provided by the manufacturer [22]. In some cases, enzyme-linked immunoassay (BMG LABTECH, Offenburg, Baden-Württemberg, Germany) was used to verify Bioplex results. Values were obtained from the linear part of the standard curve in all cases; if necessary, dilutions were made.

The spiropiperidine compound 1′-[2-[4-(Trifluoromethyl)phenyl]ethyl]-spiro [4H-3,1-benzoxazine-4,4′-peperidin]-2(1H)-one, described by Mirzadegan et al. [23] and known as RS102895, was obtained from Sigma-Aldrich (St. Louis, Missouri, USA). This compound was dissolved in dimethylsulfoxide (DMSO). For treatments with RS102895, CD14+ cells were isolated, counted and adjusted to appropriate density in medium and plated into multiwall plates. After one hour to permit cell settling, agents were added in 100 μL medium to reach the final desired concentration. Controls were treated with an equal amount of the vehicle.

### 2.2. Real-Time Polymerase Chain Reaction (RT-PCR)

The RNA was isolated from cultured cells in multi-well plates using TRI-ZOL reagent (Invitrogen, Waltham, MA, USA) as per the manufacturer’s instructions. cDNA was produced using the Improm-2 reverse transcriptase system (Promega, Madison, WI, USA) using both random primers (300 ng) and oligo dT (300 ng). RT-PCR was carried out using the Bio-Rad iQ iCycler real-time PCR system using SYBR Green 1 supermix (Bio-Rad) using primers and conditions as previously described [8,9,24,25]. We used measures of RT-PCR quality such as melt curve and polyacrylamide gel electrophoresis of PCR products after RT-PCR as previously described [24,25]. Cycle threshold (CT) for real time measures were taken according to the instructions for the BioRad i-cycler. A number of validated housekeeping genes were used in these experiments: glutaldehyde dehydrogenase (GAPDH), beta 2 microglubulin (B2M), 18S rRNA (18S), hypoxanthine-gauanine phosphoribosyl transferase (HPRT), porphobilinogen deaminase (PBDG) [26]. These housekeepers were highly correlated (R > 0.96 for all comparisons) and gave similar results but in any given experiment, one or two such housekeepers may have been used as indicated in figure legends. For real time PCR assays that were calibrated against standard amounts of target transcript, data are expressed either as mRNA copies per 10^5^ copy of GAPDH or per million copies of 18S, depending on which particular housekeeper was used in that experiment. Primers used in this study are shown in Table 1 and are derived from this study or prior work as indicated [8,9,10,24,25,26].

### 2.3. Microscopy

Osteoclast-like multinucleated giant cells were considered as cells with three or more nuclei. Two indexes were used to measure the formation of such osteoclast-like cells. Osteoclast area is the area of a microscope field occupied by osteoclast-like multinucleated giant cells as a percentage of the total area of the field. Typically, five fields are measured per culture. Osteoclast fusion index is the proportion of nuclei found within multinuclear giant cells with three or more nuclei as a percentage of the total number of nuclei in microscopy fields. Typically, between 500 and 5000 nuclei are counted to establish the fusion index. Since nuclei do not divide in osteoclasts, the fusion index is a measure of the total accumulated fusion events in the culture. Hoffman modulation contrast microscopy was used to view live cells. Cells were also fixed and stained with 4′,6-diamidino-2-phenylindole (DAPI) to visualise nuclei, tartrate resistant acid phosphatase (TRAP) to identify TRAP+ cells and rhodamine-phalloidin to identify F-actin rings as described previously [8]. We did not examine bone resorption in this study as we were interested in the formation of multinucleated giant cells rather than their mature function.

### 2.4. Statistical Analysis

For real time PCR, the difference between the cycle threshold (CT) of the gene being examined and the reference housekeeper gene was referred to as ΔCT. Data were considered on occasion as fold difference using the delta-delta CT (ΔΔCT) method. CT, ΔCT and ΔΔCT values are normally distributed parameters, and are suitable for analysis of variance (ANOVA) and *t*-tests. In all figures, error bars are standard error of the mean. Data are derived from either duplicate or triplicate cell cultures performed simultaneously. Occasionally, as described in figure legends, data are presented as relative mRNA levels, meaning that all differences are presented relative to a particular treatment used as a comparison. The Pearson’s chi-squared test was used to compare counts of nuclei within MNC and non-MNC populations. In measures of inhibition of osteoclast formation, the hypothesis is one-tailed, in that suppression is expected for osteoclast phenotypic measures. Gene expression and other phenotypic measures are correlated tests of the null hypothesis tested by linear regression against log of concentration for each parameter.

## 3. Results

### 3.1. Osteoclast-like Cells in Culture Derived from Human CD14+ Mononuclear Cells

**Figure 1:**
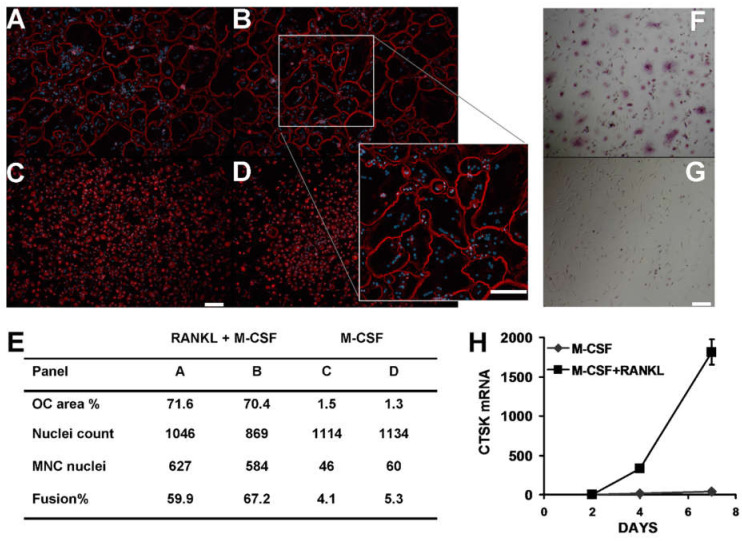
Human CD14+ mononuclear cells form multinucleated giant cells (MNC) with osteoclast features such as F-actin rings and osteoclast marker gene expression when cultured in vitro with M-CSF and RANKL. Panels (**A**,**B**) show large MNC with F-actin rings (red colour) and multiple nuclei (blue colour) identified by fluorescence micrographs in representative cultures treated with M-CSF and RANKL. Insert in B indicated by white box and grey lines shows higher magnification. Panels (**C**,**D**) are matched controls for panel (**A**,**B**), respectively, cultured with M-CSF alone. Cells were cultured for 7 days and then fixed and stained with rhodamine-phalloidin to identify F-actin rings (red colour) and counterstained to identify nuclei (DAPI, blue colour). (**E**) Table shows quantitative image analysis data derived from images (**A**–**D**), with treatment type, percentage of total micrograph field area occupied by osteoclast-like MNC with three or more nuclei (OC area %), total nuclei count, number of nuclei found within MNC, and the proportion of total nuclei found within MNC (Fusion%). (**F**) Magenta colour is TRAP stain in CD14+ cells cultured for 5 days with M-CSF and RANKL compared to M-CSF alone (panel (**G**)). Although some mononuclear cells have some minor TRAP activity in both RANKL treated cultures and control M-CSF alone, TRAP stain is more prominent within MNC. White scale bar in (**B**) insert, (**C**,**G**) is 100 microns. (**H**) Induction of osteoclast marker gene cathepsin K (CTSK mRNA) in CD14+ cells treated with M-CSF and RANKL (black squares) compared to M-CSF alone (grey diamonds). Graph data are relative to zero-time value set as 1. Error bars are smaller than the graph symbol at all times except 7 days M-CSF + RANKL (N = 3 for all data points, *p* = 1.2 × 10^−8^ and 7.8 × 10^−8^ at 4 and 7 days, respectively).

The CD14+ fraction of PBMC was isolated from fresh blood and plated directly in medium supplemented with either 25 ng/mL M-CSF or 25 ng/mL M-CSF and 40 ng/mL RANKL, in order to verify the formation of multinucleated osteoclast-like cells. Such cultures produced abundant osteoclast-like cells with prominent F-actin rings and numerous nuclei (Figure 1A,B) at 7 days. Control cultures treated with M-CSF alone produced low numbers of MNC in comparison (Figure 1C,D). The area of image occupied by osteoclast-like cells (about 70%) and the number of nuclei involved in cell fusion events indicates that many CD14+ cells in such cultures are effective precursors capable of forming multinucleated osteoclast-like cells (Figure 1E). At five days, osteoclast-like multinuclear cells were strongly positive for TRAP compared to control cells treated with M-CSF alone (Figure 1F,G). Cathepsin K mRNA (CTSK mRNA) was strongly induced by RANKL over 7 days, relative to zero time (Figure 1H). In comparison, cells treated with M-CSF alone had low levels of TRAP and CTSK (Figure 1G,H). These data confirm the osteoclast-like phenotype of such CD14+ derived cells with key markers TRAP and CTSK and with abundant multi-nucleation and prominent F-actin rings, although we did not measure bone resorption activity.

### 3.2. Effect of M-CSF and GM-CSF on MCP1 Abundance in CD14+ Mononuclear Cells

**Figure 2:**
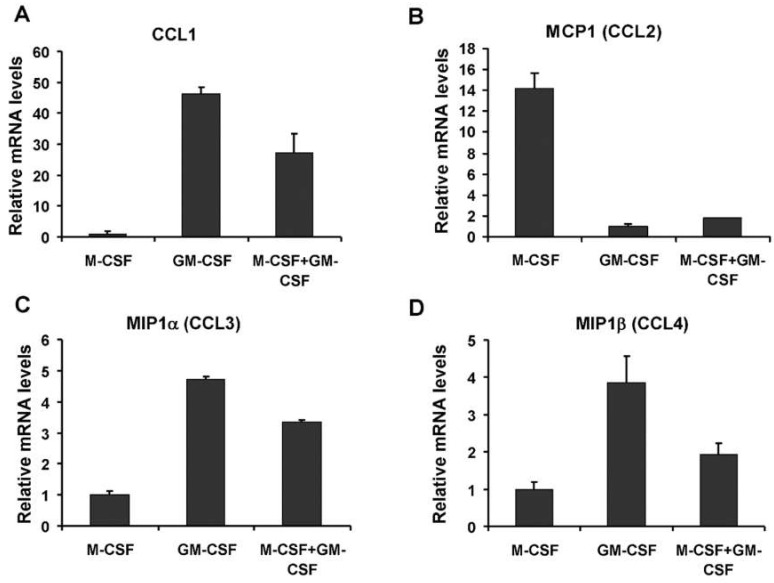
Relative gene expression of chemokines varies according to culture conditions in CD14+ human monocytes. (**A**) GM-CSF treatment induces CCL1 relative to M-CSF treatment (*p* = 0.0002). (**B**) M-CSF treatment induces MCP1 (CCL2) relative to GM-CSF treatment (*p* = 0.003). (**C**) MIP1α (CCL3) is induced by GM-CSF (*p* = 0.0001), as is MIP1β (CCL4) shown in graph (**D**) (*p* = 0.03). M-CSF + GM-CSF means simultaneous treatment with both agents. Each data point is derived from duplicate identical cultures.

Human CD14+ mononuclear cells were isolated from fresh blood and plated in medium supplemented with either M-CSF, GM-CSF or a combination of M-CSF and GM-CSF to test the effect of culture pre-treatment on the expression of chemokines. Relative mRNA levels for various chemokines were determined. These data show that GM-CSF induces the expression of chemokines CCL1, CCL3 and CCL4 while M-CSF induces expression of MCP1 (Figure 2A–D). CCL1 mRNA was about 45-fold more abundant in GM-CSF treated cells compared to M-CSF treated cells (Figure 2A). Simultaneous treatment with M-CSF and GM-CSF produced a CCL1 mRNA level roughly intermediate between that found in treatment of M-CSF alone and GM-CSF alone (Figure 2A). MCP1 mRNA was 14-fold more abundant in M-CSF compared to GM-CSF treated cells (Figure 2B, *p* = 0.003). MCP1 mRNA levels were not intermediate in combined M-CSF and GM-CSF treatments, showing that GM-CSF suppression of MCP1 still occurs in the presence of M-CSF (Figure 2B), at least at these concentrations. Members of the MIP1 family, CCL3 (MIP1α) and CCL4 (MIP1β) were induced by GM-CSF treatment relative to M-CSF treatment to varying degrees (Figure 2C,D, *p* = 0.0001 and *p* = 0.03, respectively). For CCL1, CCL3 and CCL4, the combined treatment with GM-CSF plus M-CSF, resulted in intermediate mRNA levels, suggesting some suppression of GM-CSF induction, relative to GM-CSF alone treated cells.

**Figure 3:**
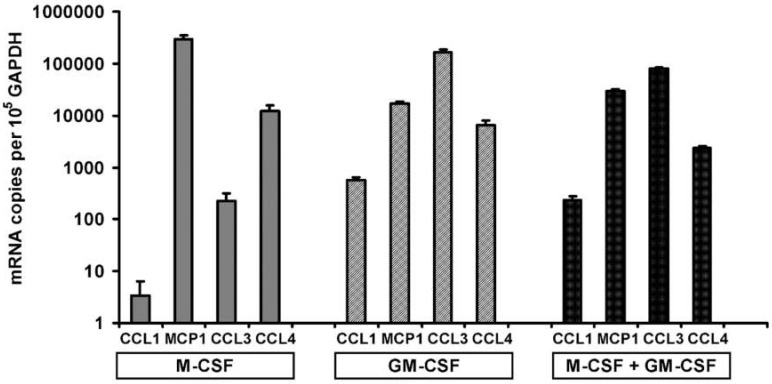
Chemokine mRNA transcript levels according to culture conditions. The data are derived from calibrated quantitative RT-PCR. The data are presented as transcript copies per 10^5^ GAPDH mRNA transcripts on a logarithmic scale. MCP1 mRNA in M-CSF treated cells was the most abundant of measured transcripts, being estimated at 3 MCP1 per GAPDH, which also corresponds to 52,000 ± 11,000 MCP1 transcripts per million 18S RNA molecules. M-CSF treated samples are indicated by grey shading, GM-CSF treated samples indicated by cross hatching and combined M-CSF plus GM-CSF (M-CSF + GM-CSF) treated samples are indicated by black shading. ANOVA *p* values for treatment effect for each gene are as follows: CCL1 *p* = 0.004, MCP1 *p* = 0.001, CCL3 *p* = 0.002 and CCL4 *p* = 0.02.

The chemokine CCL5 (also known as RANTES) was expressed at very low levels in all experiments (data not shown). Although CCL1 mRNA was about 45 times more abundant in GM-CSF treated cells compared to M-CSF treated cells (Figure 2A), the absolute amount of CCL1 mRNA was the lowest of the chemokines measured (Figure 3). MIP1α (CCL3) and MIP1β (CCL4) are similar chemokines and are ligands of the receptors CCR1 and CCR5. When absolute amounts of chemokine mRNAs are considered, MCP1 (CCL2) and MIP1α (CCL3) were considered abundant transcripts in osteoclast precursors (Figure 3). In M-CSF treated cells, MCP1 mRNA was about three times more abundant than the GAPDH housekeeper mRNA (Figure 3). MCP1 mRNA was more than 1300-fold abundant than MIP1α (CCL3) mRNA in M-CSF treated cells and about 24 times more abundant than the combined amounts of both MIP1 family chemokines (CCL3 and CCL4). Conversely, in GM-CSF treated cells, the MIP1 family chemokines were collectively 10 times more abundant than MCP1. Since MCP1 mRNA is still present in cells treated with GM-CSF, we may expect MCP1 protein to be present, although reduced in the culture media of cells treated with GM-CSF.

The data suggest, in CD14+ monocytes, that: (1) M-CSF induces MCP1 and suppresses CCL1 and the MIP1 family members, MIP1α (CCL3) and MIP1β (CCL4); and (2) that GM-CSF suppresses MCP1 while inducing CCL1, CCL3 and CCL4. Therefore M-CSF and GM-CSF compete to produce a different chemokine outcome in human osteoclast precursor cultures.

### 3.3. Expression of Chemokine Receptors

**Figure 4:**
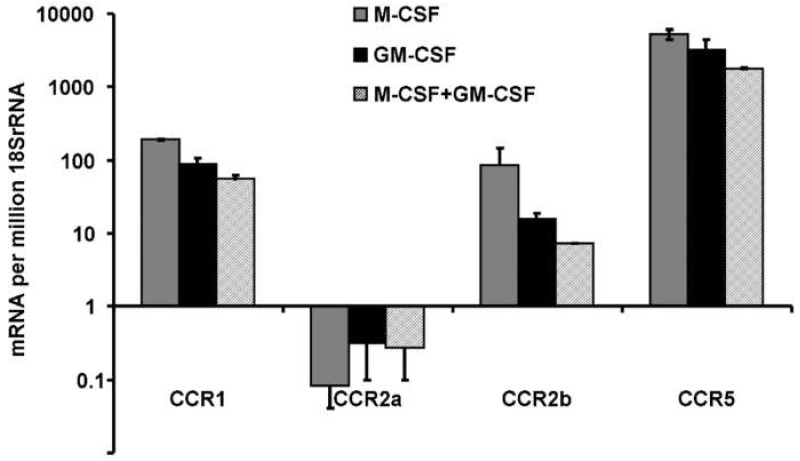
Chemokine receptor mRNA transcript levels according to culture conditions. CD14+ human mononuclear cells were cultured in medium containing either M-CSF, GM-CSF or M-CSF plus GM-CSF. Calibrated real time PCR assays indicate that the rank order of mRNA abundance is CCR5 > CCR1 > CCR2b. The CCR2a receptor variant is much less abundant than CCR2b (*p* = 0.001). Except for CCR2a, transcript levels are highest for each gene in M-CSF treated cells and lowest in the combination of M-CSF and GM-CSF (*p* = 0.008, *p* = 0.015 for CCR1 and CCR2b, respectively). CCR5 expression followed the same trend but the overall ANOVA was not significant (*p* = 0.08). Legend indicates treatments.

The expression of chemokine receptors from CCR1 through to CCR8 was assessed using RT-PCR in CD14+ human mononuclear cells pre-treated with either M-CSF, GM-CSF or M-CSF combined with GM-CSF. Chemokine receptors CCR3, 4, 6, 7 and 8 had very low transcript abundance and will not be considered further. For those receptors where assays were calibrated (CCR1, CCR2 and CCR5), assays suggest a rank order of mRNA abundance of CCR5 > CCR1 > CCR2b mRNA transcript in this experiment (Figure 4). Although CCR5 transcript is abundant, CCL5 mRNA was rare. The CCR2b isoform of CCR2 mRNA was more abundant than CCR2a. M-CSF pre-treatment resulted in the highest levels of these receptor mRNAs, and all were repressed somewhat by GM-CSF pre-treatment, either alone or with M-CSF. Since protein levels of receptors were not measured, the interpretation of such a rank order of mRNA abundance cannot extend to the functional receptor.

### 3.4. Pre-Treatment and Post-Treatment Culture Conditions and Chemokine mRNA Levels

In this series of experiments, CD14+ cells were isolated from peripheral blood and then stimulated to form osteoclasts using RANKL in the presence of M-CSF after three different pre-treatments. Cells were pre-treated for five days with either M-CSF, GM-CSF (25 ng/mL) or M-CSF plus GM-CSF (both at 25 ng/mL) prior to media change then subsequent treatment under conditions to generate macrophage-like cells (M-CSF alone) or osteoclast-like cells (RANKL plus M-CSF). A zero-time sample was taken, representing the state of the cells immediately after the 5 day pre-treatment. The effect of pre-treatment of cells on gene expression was followed day 1, 3 and 5 after media change (Supplementary Figure S1A–F). CCL1 mRNA levels were of low abundance in all pre-treatments with GM-CSF (Figure S1A). In cells pre-treated with M-CSF alone, CCL1 showed detectable expression at day 1 in both post-treatments (M-CSF and M-CSF RANKL). Likewise, MCP1 (CCL2) expression was suppressed in all cultures pre-treated with GM-CSF regardless of post-treatment or the time (Figure S1B). However, MCP1 mRNA was abundant at the day zero-time point after pre-treatment with M-CSF. A decline in MCP1 mRNA was seen from day 0 to day 5 in both M-CSF and M-CSF plus RANKL post-treatments. However, despite the decline in MCP1 mRNA over that time, the level at the end of the post-treatment period was still higher than that of the other tested chemokine transcripts.

In M-CSF pre-treated cells, MIP1α (CCL3) mRNA was of low abundance at zero time (Figure S1C), but showed minor increased expression at day 1, 3 and 5 in the fresh medium containing M-CSF. This expression pattern occurred also in cells post-treated with M-CSF and RANKL. MIP1α (CCL3) mRNA was most abundant at the zero time of GM-CSF pre-treatment (Figure S1C). In post-treatment with M-CSF and M-CSF plus RANKL, similar variable patterns were observed: at day 1, a drop in expression occurred, consistent with M-CSF treatment not sustaining gene expression of MIP1α (CCL3), and at other times, expression was low and variable. Similarly, MIP1β (CCL4) mRNA abundance was also lowest in M-CSF pre-treated cells and more abundant in GM-CSF treated cells (Figure S1D). Despite having overall low abundance, MIP1β (CCL4) mRNA levels showed a strong induction effect when pre-treatment medium containing M-CSF was replaced with an identically prepared new medium. CCL5 mRNA (Figure S1E) was of very low abundance in all samples and was only detectable after day 1 in cells pre-treated with M-CSF. CCR1 mRNA abundance was also examined, being the primary receptor for MIP1 family members and already implicated in osteoclast biology (Figure S1F). CCR1 transcript levels were highest at zero time in M-CSF pre-treated cells and showed initial suppression on day 1 in fresh media but with a pattern of high variability in all cultures that limits interpretation of the data. CCR2 and CCR5 were not examined in this experiment.

### 3.5. Pre-Treatment Culture Conditions and MCP1 Protein Levels

**Figure 5:**
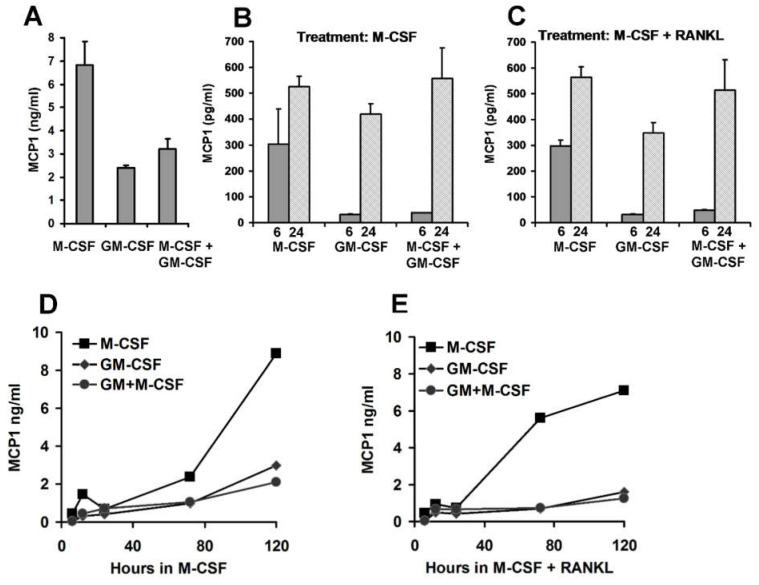
MCP1 protein accumulation in media of CD14+ mononuclear cells cultured for 5 days in M-CSF, GM-CSF or M-CSF and GM-CSF combined (M-CSF + GM-CSF). (**A**) CD14+ mononuclear cells treated for 5 days with M-CSF alone showed higher MCP1 accumulation compared to cells treated with GM-CSF or M-CSF + GM-CSF (*p* = 0.016 and 0.028, respectively). (**B**) Suppression of MCP1 production in cultures pre-treated with GM-CSF was evident at six hours (grey columns marked 6) in cultures post-treated with M-CSF (Graph (**B**), *p* = 0.03) and M-CSF and RANKL (Graph (**C**), *p* = 0.00009). At 24 h post medium change (columns marked 24) MCP1 was not significantly different across culture conditions (ANOVA *p* = 0.06). (**C**) Identical cultures to those in (**B**) were also treated with M-CSF and RANKL after the three different pre-treatments. (**D**) Although MCP1 levels were similar at 24 h, suppression of MCP1 protein accumulation by pre-treatment with GM-CSF was evident for up to five days after removal of GM-CSF and post-treatment with either M-CSF alone (**D**) or M-CSF and RANKL. (**E**) Pre-treatment conditions are signified by symbols as marked. For graphs (**B**,**C**) data points are derived from identical duplicate cultures harvested at that time. For (**D**,**E**), each of the six data lines is from a single culture sampled repeatedly through time.

Chemokine protein levels were assessed in similar cell experiments. Cells were cultured as above with pre-treatment with either M-CSF, GM-CSF or GM-CSF plus M-CSF combined treatment. After five days, MCP1 accumulated to around 7 ng/mL in the medium of M-CSF treated cells, but to a lesser extent in cells pre-treated with GM-CSF (Figure 5A). The medium of pre-treated cells was thoroughly but gently removed without disturbing the cells and then the cells were cultured in fresh medium with either M-CSF alone or the standard combination of M-CSF and RANKL that is used to induce osteoclast-like cells. MCP1 protein was assayed at 6 and 24 h post media change (Figure 5B). The cell culture that had been pre-treated with M-CSF produced around 300 pg/mL of MCP1 in medium within six hours of culture. Cultures pre-treated with GM-CSF or GM-CSF and M-CSF together produced less MCP1 (Figure 5B,C). Within 24 h of culture, MCP1 levels in culture media were around 500 pg/mL in M-CSF pre-treated cultures (Figure 5B). After 24 h in fresh medium, the effects of pre-treatment with GM-CSF were less apparent and MCP1 levels were similar in culture media in all post-treated cultures. Despite this fact, over a longer time scale, the suppressive effect of GM-CSF pre-treatment on MCP1 accumulation in culture media persisted for at least five days after removal of GM-CSF (Figure 5D,E).

### 3.6. Chemokine Accumulation in Media of CD14+ Human Mononuclear Cells in Culture

**Figure 6:**
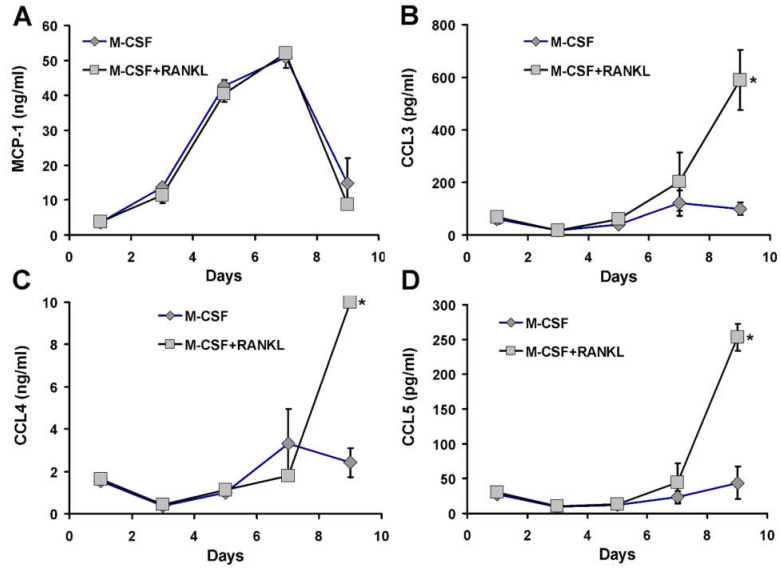
Chemokine accumulation in culture media over time. CD14+ human mononuclear cells were cultured with M-CSF (grey diamonds) or M-CSF and RANKL (grey squares) and media assayed for chemokine accumulation. (**A**) MCP1 accumulates over time to over 50 ng/mL within 7 days of culture. MCP1 then declines in culture media at 9 days when media is practically exhausted, and cells begin to die. (**B**) CCL3 (MIP1α) is at picogram per millilitre concentrations throughout both macrophage (M-CSF) or osteoclast (M-CSF + RANKL) culture and reaches a peak after the maximal formation of osteoclasts (at day 5 to 7 in such cultures). (**C**) CCL4 (MIP1β) concentration is in the low ng/mL range throughout the cultures and similar to CCL3, increasing towards the end of the culture of RANKL treated cells. (**D**) CCL5 (also known as RANTES) is detected at low pg/mL concentrations and in a manner similar to CCL3 and CCL4, rises only at the end of the culture period in the RANKL treated cells. Asterisk indicates a RANKL-related value that is significantly different from that of the time matched M-CSF treated cell value (CCL3 *p* = 0.006, CCL4 *p* = 0.0001; CCL5, *p* = 0.001). Data derived from triplicate simultaneous cultures sampled repeatedly.

We determined the relative rank order of abundance of MCP1 and other chemokines secreted in media by CD14+ cells in culture. Furthermore, we tested whether there was any difference in chemokine accumulation between M-CSF alone and M-CSF plus RANKL treatment. These cultures were set up so that sufficient media were present to permit repeated sampling of 100 μL aliquots and that media did not require changing during the assay period. CD14+ cells were purified as before and plated in media supplemented with either M-CSF alone or the standard combination of M-CSF plus RANKL. Aliquots were taken at third-daily intervals and assayed for chemokines. MCP1 was clearly very abundant in culture media, with levels up to 50 ng/mL (Figure 6A), exceeding all other measured chemokines (Figure 6B–D). There was little difference in the time course of accumulation of MCP1 in M-CSF treatment compared to M-CSF and RANKL treatment (Figure 6A). After one day in culture, the concentration of MCP1 was approaching 1 ng/mL and within 3 days was around 12 ng/mL. In such human cell cultures, osteoclast-like cells form between 4 and 7 days of culture (Figure 1). Although we did not assay every day, MCP1 levels were around 50 ng/mL at 7 days of culture. Interestingly, in similar experiments, peak MCP1 mRNA abundance occurred much earlier and appeared to be declining even after the first day in similarly treated cultures (Figure S1B), although this notion requires comparing two different experiments. A negative feedback control of MCP1 mRNA expression would indeed result in the peak protein accumulation in media corresponding with more modest mRNA levels, especially if MCP1 protein were stable in media. In other words, high early mRNA levels would drive increasing accumulation of MCP1, leading to feedback decrease in mRNA levels.

In contrast to MCP1, other assayed chemokines accumulated more slowly and to a much lower concentration (Figure 6B–D). Most significantly, at three days when osteoclast precursors are proliferating, MCP1 protein in media (12.5 ng/mL) was about 29 times more abundant than the combined amount of measured CCR1 ligands (MIP1α, MIP1β and CCL5). MIP1α (CCL3) showed a steady increase to around 700 pg/mL at day 9 with a differential between M-CSF and RANKL treated cells (Figure 6B). MIP1β (CCL4) was more abundant than MIP1α (CCL3), being in the ng/mL rather than pg/mL range, reaching 10 ng/mL at day 9 in RANKL treated cultures (Figure 6C).

RANKL had a specific effect to increase MIP1β between 7 and 9 days, coinciding with a drop in MCP1 concentration. In contrast, CCL5 accumulated to low levels, reaching around 270 pg/mL at day 9 (Figure 6D). The peak of MCP1 production in these cultures coincides with the peak of osteoclast-like cell formation (days 5 to 7), whereas the peak accumulation of MIP1α, MIP1β and CCL5 occurred after osteoclast formation and indeed when substantial osteoclast apoptosis was evident by microscopic observation. Other factors assayed in the 27-plex Biorad bioplex assay were either undetectable or at very low levels, except interleukin receptor antagonist (IL1RA) which reached 8 ng/mL at day 9 and CXCL8 (also called interleukin 8) which was between 5 and 8 ng/mL for the entire period of the experiment (data not shown). Neither IL1RA nor CXCL8 showed RANKL specific induction.

The data shows that MCP1 protein is abundant in media from cultured CD14+ cells, greatly exceeding that of other chemokines assayed. RANKL did not alter MCP1 accumulation but had a late effect on accumulation of CCL3, CCL4 and CCL5.

### 3.7. Effect of CCR2 Antagonist on Human Osteoclast Formation and Function

**Figure 7:**
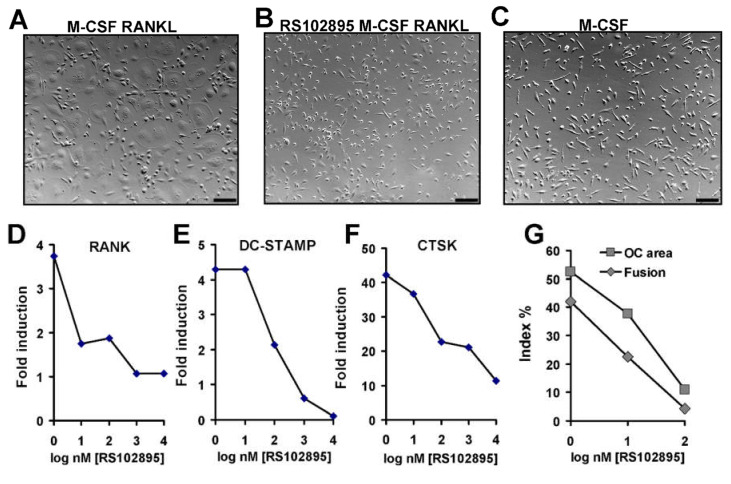
The effect of CCR2 antagonist RS102895 on human osteoclast formation analysed by microscopy and gene expression studies. (**A**) Normal osteoclasts formed via treatment with M-CSF and RANKL. Hoffman modulation contrast optics; note the presence of giant cells. (**B**) Inhibition of M-CSF and RANKL mediated osteoclast formation by the continual presence of RS102895 (500 nM). (**C**) Cells treated with M-CSF alone have an abundance and an appearance identical to cells in panel (**B**). Graphs (**D**–**F**) Gene expression studies using RT-PCR on RS102895 treated cells show suppression of RANKL mediated induction of key osteoclast marker genes. RS102895 concentrations are given as logarithm of nM. Fold induction is the gene expression relative to control cells, treated with M-CSF alone. (**D**) Suppression of RANK, the receptor for RANKL. (**E**) Suppression of DC-STAMP, a cell fusion mediator. (**F**) Suppression of cathepsin K (CTSK), a proteolytic enzyme used as a mature osteoclast functional marker. (**G**) Suppression of osteoclast indexes observed by microscopy. Osteoclast area index (OC area, grey boxes on graph) is the percentage area of a standard microscopy field occupied by osteoclasts. Fusion index (grey diamonds on graph) is the ratio of the total count of nuclei found within osteoclasts as a percentage of the total count of all nuclei in micrographs. Data in graph (**G**) are derived from counting a total of 5766 nuclei by microscopy. One-tailed *p* values for each individual regression are as follows: RANK, *p* = 0.027; DC-STAMP, *p* = 0.004; CTSK, *p* = 0.003, Fusion, *p* = 0.005; OC Area, *p* = 0.007. Scale bar is 100 microns.

The compound RS102895 is a small molecule antagonist of chemokine receptor CCR2 with IC50 values reported as 360 nM and 17.8 μM for CCR2b and CCR1, respectively [22]. The amount of MCP1 produced in human CD14+ mononuclear cell cultures (up to 50 ng/mL) corresponds to about 5.8 nM MCP1 in media. CD14+ human mononuclear cells were isolated and cultured with continuous exposure to various concentrations of RS102895 in the presence of RANKL and M-CSF for five days before harvest. Receptor activator of NF-κB (RANK), cathepsin K (CTSK) and dendrocyte expressed seven transmembrane protein (DC-STAMP) were chosen as gene expression markers related to osteoclast differentiation, function and cell fusion, respectively, as described previously [17,18]. At 500 nM, RS102895 caused a total inhibition of human osteoclast-like cell formation (Figure 7A,B) but did not cause cell death relative to M-CSF alone treated cells (Figure 7C). RS102895 showed a dose-response of inhibition of gene expression of key osteoclast marker genes with a half maximal concentration of approximately 100 nM (Figure 7D–F). A dose responsive repression of osteoclast formation by RS102895 was observed for the osteoclast area and osteoclast fusion indexes (Figure 7G). These data suggest that MCP1 signalling via CCR2 is necessary for human osteoclast precursor differentiation from human CD14+ mononuclear cells.

## 4. Discussion

The key observation in this study is the surprisingly large amount of MCP1 made by human osteoclast precursor cultures during treatment with M-CSF. These data show that conditioned media from human osteoclasts may contain up to 50 ng/mL MCP1. Prior work suggested that RANKL was responsible for MCP1 increase; however, this work using CD14+ human cells suggests that M-CSF is the primary driver of MCP1 expression. Several authors using human, mouse and rat cell systems and different culture conditions, report that adding exogenous MCP1 (usually between 50 to 100 ng/mL) to osteoclast cultures leads to larger, more abundant osteoclasts with higher nuclear counts [8,9,10,12,13,14]. On a simple level, the reason that supplementation of MCP1 to osteoclast cultures increases osteoclast formation may be that it replaces MCP1 that accumulates naturally and is lost in media change. Therefore, routine supplementation with MCP1 in media of osteoclast cultures should permit production of larger osteoclasts in a shorter period.

Of human osteoclast models, crude PBMC preparations isolated from blood by density gradient centrifugation are the easiest to prepare but are a mixed culture of many cell types including some that suppress osteoclast formation, such as T-cells. Colony-forming unit granulocyte-macrophage (CFU-GM) and colony-forming unit macrophage (CFU-M) have high osteoclastic potential but are rather difficult to prepare, requiring cloning in methocult of hematopoetic stem-like cells from umbilical cord blood [27]. Such cells are cultured with a mixture of cytokines that promote stem characteristics before exposure to RANKL. CD14+ mononuclear cells are much more convenient to prepare than CFU-M or CFU-GM and are a better source for human osteoclast progenitors than PBMC. CD14+ cells form abundant osteoclast-like cells in culture within five to seven days and are therefore the best compromise between ease of isolation and the number of osteoclast progenitors in the preparation.

We did not observe any meaningful difference in any chemokine concentration or gene expression comparing M-CSF treated cultures with M-CSF and RANKL treated cultures. Any differences that were observed occurred at 9 days of culture, after osteoclast formation, and coincident with the cultures reaching the end of their capacity to grow. Osteoclasts have limited lifespans and apoptotic material accumulates in cultures as osteoclasts expire. The apparent RANKL difference in MIP1α (CCL3), MIP1β (CCL4) and RANTES (CCL5) protein concentrations at day nine (see Figure 6) may be a RANKL specific effect or, alternatively, may be due to responses of mononuclear macrophage-like cells to osteoclast apoptotic material rather than a specific RANKL late timed effect. Regardless, the timing of such chemokine increases occurs well after the peak formation of osteoclast-like cells and are therefore of enigmatic functional relevance.

How the MCP1 enhancement of osteoclast formation occurs is not yet understood, but may relate to either precursor expansion, cell fusion or osteoclast life span. MCP1 protein in culture medium reaches ng/mL levels quickly in the presence of M-CSF. The lack of difference in MCP1 concentrations between M-CSF and M-CSF plus RANKL treated cultures suggests that the large amount of MCP1 produced is a component of macrophage lineage differentiation under the influence of M-CSF. In other words, more mononuclear osteoclast precursor cells might differentiate or survive better in the presence of MCP1 induced by M-CSF. Osteoclast numbers are increased on treatment with MCP1 and enhanced bone resorption has been observed with MCP1 supplementation [8,9,10]. Such outcomes would also flow from increased survival of legitimate osteoclast precursors and such an effect on osteoclast precursors would generate all the biological effects observed so far and is the simplest current model of action of MCP1. Further work is needed to test these alternatives.

The CCR2 antagonist RS102895 blocked CCR2 mediated early effects in vivo in a mouse fracture healing model resulting in delayed healing and much lower numbers of TRAP+ osteoclasts in the fracture site [28]. Our prior in vitro studies showed blockade of MCP1 reduces human osteoclast formation using both neutralising anti-MCP1 antibody and 7ND (the dominant negative MCP1) [9,10,11]. Likewise, with RS102895, we applied these agents continuously from zero time since MCP1 rises rapidly, and we suspected that MCP1 acts on early differentiation events. RANK is the cognate receptor for RANKL, is found on osteoclast precursors and is needed for osteoclast formation. DC-STAMP is a cell surface protein important for the fusion of osteoclasts and foreign body giant cells. RS102895 strongly reduced the expression of both DC-STAMP and cathepsin K, a marker of final osteoclast function. Both RANK and DC-STAMP are required for early stages of osteoclast formation and, if osteoclast do not form, a reduction in a late marker such as cathepsin K is expected. The data are consistent with blockade of early stages of osteoclast formation, but we cannot distinguish between blocking the differentiation of osteoclast-like cells from precursors, or the inhibition of expansion of such precursors in culture. Inhibition of MCP1 signalling by several different agents results in a decrease in human osteoclast-like cell formation and merits further work.

## Figures and Tables

**Table 1:** Primers used in this study.

Gene	Forward	Reverse	Size	Reference
CCL1	GACACAGTTGGATGGGTTCA	CCTCTGTGACCTAGCAAAAG	174	This study
MCP1(CCL2)	TCGCGAGCTATAGAAGAATCA	TGTTCAAGTCTTCGGAGTTTG	161	[9]
CCL3	CTATGGACTGGTTGTTGCCA	AGGGGAACTCTCAGAGCAAA	140	[24]
CCL4	TCCATGAGACACATCTCCTC	GCAACAGCAGAGAAACAGTG	186	This study
CCL5	GAGCTTCTGAGGCGCTGCT	TCTAGAGGCATGCTGACTTC	112	[24]
CCR1	TTCCTGTTCACCCATGAGTG	AAGGGGAGCCATTTAACCAG	191	[24]
CCR2a	CATAGCTCTTGGCTGTAGGA	GTGAAGCCAGACGTGTGATT	224	[24]
CCR2b	AACAAACACGCCTTCCACTG	GTCAAAGTCTCTACCCACAG	259	[24]
CCR3	ACCCTACAATGTGGCTATCC	TTCATGCAGCAGTGGGAGTA	132	This study
CCR4	CTTATGGGGTCATCACCAGT	AGTAGGTATGGTTGCGCTCA	105	[24]
CCR5	ACCAAGCTATGCAGGTGACA	GAACAGCATTTGCAGAAGCG	148	[25]
CCR6	GGACAGCTGGAATTATGCTG	CCCATGACAGTACCTTCCTA	208	This study
CCR7	ACGCAACTTTGAGCGCAACA	TTGCTTACTGAGCTCACAGG	151	This study
CCR8	GTCCCATTCAACGTGGTTCT	AGCTCTCCCTAGGCATTTGT	250	This study
CTSK	TGAGGCTTCTCTTGGTGTCCATAC	AAAGGGTGTCATTACTGCGGG	134	[8]
TRAP	GACCACCTTGGCAATGTCTCTG	TGGCTGAGGAAGTCATCTGAGTTG	176	[8]
RANK	CAGAACTAAGCTCAGTATGTGA	GAATGCCAAGCTGCAGCAAC	121	[9]
DC-STAMP	AGACCTGGGTTCCTCTCAGTGTTAT	GTTGGTGCGATGTGGCTGAGG	326	This study
GAPDH	ACAGTCCATGCCATCACTGCC	GCCTGCTTCACCACCTTCTTG	266	[26]
18S rRNA	CTTAGAGGGACAAGTGGCG	ACGCTGAGCCAGTCAGTGTA	107	[9]

All sequences are 5′ to 3′. Size of the PCR product is in base pairs. Reference means as follows: this study or [8,9,24,25,26].

## Data Availability

The data are available on request.

## Supplementary Materials

The following are available online at https://www.mdpi.com/article/10.3390/life12060789/s1, Figure S1: Chemokine mRNA transcript changes according to culture conditions.
