# Supplementary material for: Monocyte Chemotactic Protein-1 (MCP1) Accumulation in Human Osteoclast Precursor Cultures
Source: Life (Basel). 2022 May 26;12(6):789. doi: 10.3390/life12060789 (PMC9224710; doi:10.3390/life12060789)
Supplement: Supplementary file 1 [file life-12-00789-s001.zip › life-1662653-supplementary.pdf]

life

MDPI

Article

Monocyte Chemotactic Protein-1 (MCP1) Accumulation in Human Osteoclast Precursor Cultures

Nigel A. Morrison [1,*] and Mark R. Forwood [2]

[1] School of Medical Science, Griffith University, Queensland, Australia; N.Morrison@griffith.edu.au
[2] School of Medical Science, Griffith University, Queensland, Australia; M.Forwood@griffith.edu.au
* Correspondence: N.Morrison@griffith.edu.au

Supplementary Materials

Citation: Morrison, N.A.; Forwood, M.R. Monocyte Chemotactic Protein-1 (MCP1) Accumulation in Human Osteoclast Precursor Cultures. *Life* **2022**, *12*, 789. https://doi.org/10.3390/life12060789

Academic Editor: Christina Piperi

Received: 17 March 2022
Accepted: 23 May 2022
Published: 26 May 2022

Publisher's Note: MDPI stays neutral with regard to jurisdictional claims in published maps and institutional affiliations.

Copyright: © 2022 by the authors. Licensee MDPI, Basel, Switzerland. This article is an open access article distributed under the terms and conditions of the Creative Commons Attribution (CC BY) license (https://creativecommons.org/licenses/by/4.0/).

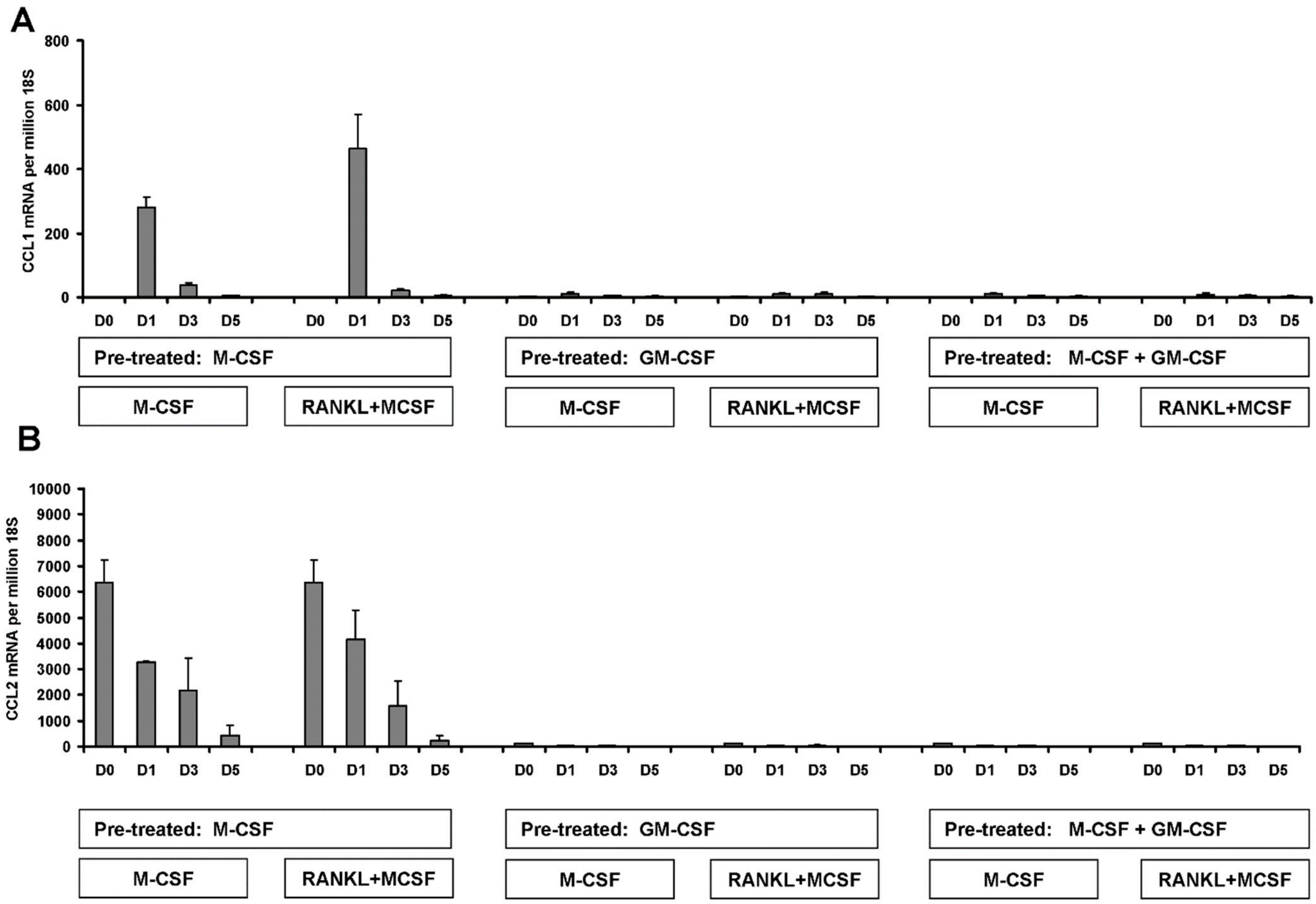
A
CCL1 mRNA per million 18S
800
600
400
200
0
D0 D1 D3 D5 D0 D1 D3 D5 D0 D1 D3 D5 D0 D1 D3 D5 D0 D1 D3 D5 D0 D1 D3 D5
Pre-treated: M-CSF
Pre-treated: GM-CSF
Pre-treated: M-CSF + GM-CSF
M-CSF
RANKL+MCSF
M-CSF
RANKL+MCSF
M-CSF
RANKL+MCSF
B
CCL2 mRNA per million 18S
10000
9000
8000
7000
6000
5000
4000
3000
2000
1000
0
D0 D1 D3 D5 D0 D1 D3 D5 D0 D1 D3 D5 D0 D1 D3 D5 D0 D1 D3 D5 D0 D1 D3 D5
Pre-treated: M-CSF
Pre-treated: GM-CSF
Pre-treated: M-CSF + GM-CSF
M-CSF
RANKL+MCSF
M-CSF
RANKL+MCSF
M-CSF
RANKL+MCSF

C

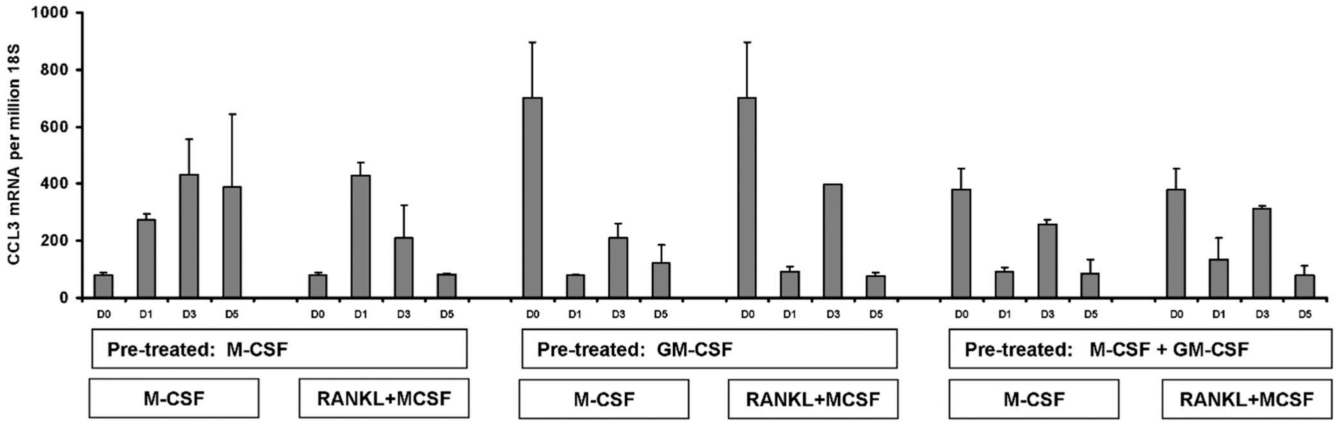
CCL3 mRNA per million 18S
1000
800
600
400
200
0
D0 D1 D3 D5 D0 D1 D3 D5 D0 D1 D3 D5 D0 D1 D3 D5 D0 D1 D3 D5 D0 D1 D3 D5
Pre-treated: M-CSF
Pre-treated: GM-CSF
Pre-treated: M-CSF + GM-CSF
M-CSF
RANKL+MCSF
M-CSF
RANKL+MCSF
M-CSF
RANKL+MCSF

D

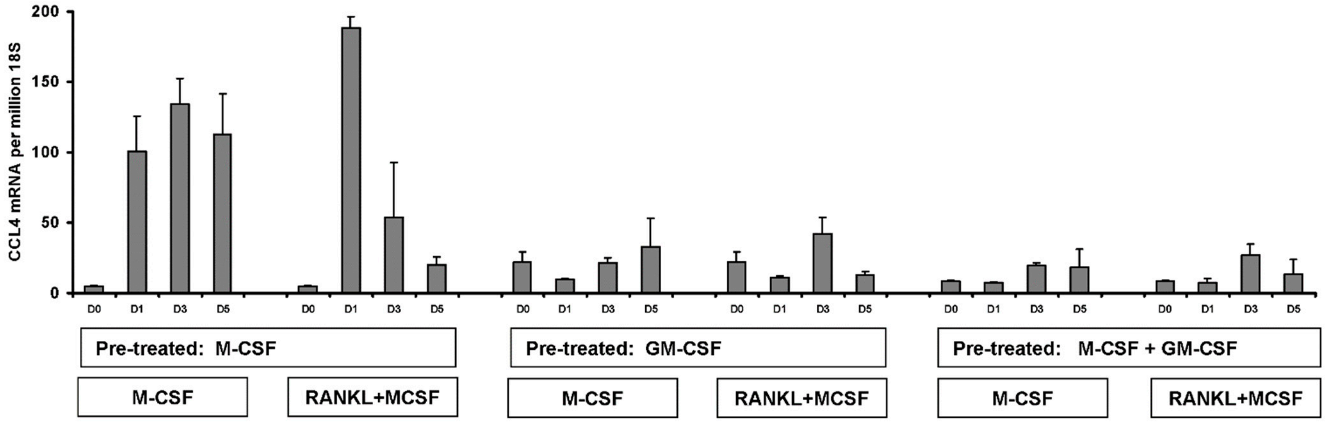
CCL4 mRNA per million 18S
200
150
100
50
0
D0 D1 D3 D5 D0 D1 D3 D5 D0 D1 D3 D5 D0 D1 D3 D5 D0 D1 D3 D5 D0 D1 D3 D5
Pre-treated: M-CSF
Pre-treated: GM-CSF
Pre-treated: M-CSF + GM-CSF
M-CSF
RANKL+MCSF
M-CSF
RANKL+MCSF
M-CSF
RANKL+MCSF

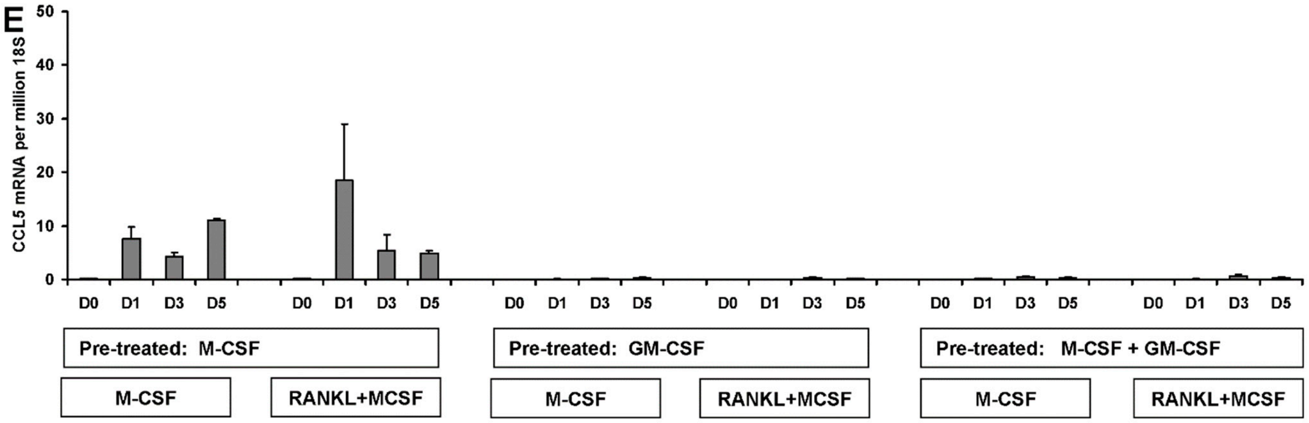

F

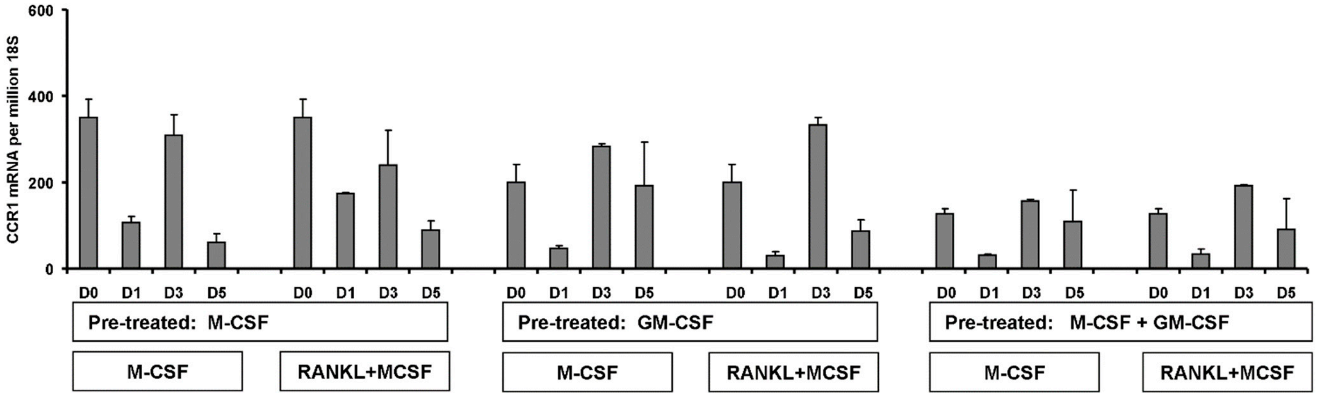

Figure S1. Chemokine mRNA transcript changes according to culture conditions. Each graph shows the outcome of six different conditions, with gene expression measured at zero time (D0), and then at days 1, 3 and 5 (D1, D3 and D5, respectively). Pre-treatment for 5 days is indicated in the first row of boxes containing the legend "Pre-treated". Post treatment is indicated in the second set of boxes labelled either M-CSF or RANKL+M-CSF. **A**. CCL1 mRNA levels are very low except at day one after media change and only in those cultures pre-treated with M-CSF. **B**. MCP1 (CCL2) mRNA transcript levels are the highest of all assayed genes and are substantially suppressed by GM-CSF pre-treatment (zero time). High MCP1 mRNA levels in M-CSF pre-treated cells decline steadily over time. In GM-CSF pre-treated cells, no recovery of MCP1 mRNA levels was observed when cells were cultured in M-CSF alone or M-CSF in combination with RANKL. **C.** CCL3 (MIP1α) mRNA levels are quite different at zero time (D0) according to pre-treatment with highest levels in GM-CSF treated cells. In cells pre-treated with M-CSF, an increase in expression is observed at days 1, 3 and 5 in both post-treatments of M-CSF and MCSF and RANKL. In GM-CSF pre-treated cells, CCL3 mRNA levels drop after day zero. **D.** CCL4 (MIP1β) has very low transcript levels at day zero (immediately after the pre-treatment period). In M-CSF post-treatment cultures and M-CSF and RANKL post-treatment cultures, CCL4 (MIP1β) shows induced gene expression effect in a pattern similar to that of CCL3 (MIP1α). **E.** CCL5 (RANTES) mRNA levels are extremely low with highest levels in cultures pre-treated with M-CSF. **F.** CCR1 mRNA transcript levels are not particularly altered and appear to vary in all cultures.
